# Artificial Intelligence in Interventional Radiology: A Literature Review and Future Perspectives

**DOI:** 10.1155/2019/6153041

**Published:** 2019-11-03

**Authors:** Roberto Iezzi, S. N. Goldberg, B. Merlino, A. Posa, V. Valentini, R. Manfredi

**Affiliations:** ^1^Fondazione Policlinico Universitario A. Gemelli IRCCS, UOC di Radiologia, Dipartimento di Diagnostica per Immagini, Radioterapia Oncologica ed Ematologia, Roma, Italy; ^2^Università Cattolica del Sacro Cuore, Istituto di Radiologia, Roma, Italy; ^3^Department of Radiology, Hadassah Hebrew University Medical Center, Jerusalem, Israel; ^4^Department of Radiology, AFaR-IRCCS Fatebenefratelli Hospital Foundation for Health Research and Education, via di Ponte Quattro Capi 39, 00186 Roma, Italy; ^5^Fondazione Policlinico Universitario A. Gemelli IRCCS, UOC di Radioterapia Oncologica, Dipartimento di Diagnostica per Immagini, Radioterapia Oncologica ed Ematologia, Roma, Italy; ^6^Università Cattolica del Sacro Cuore, Istituto di Radioterapia Oncologica, Roma, Italy

## Abstract

The term “artificial intelligence” (AI) includes computational algorithms that can perform tasks considered typical of human intelligence, with partial to complete autonomy, to produce new beneficial outputs from specific inputs. The development of AI is largely based on the introduction of artificial neural networks (ANN) that allowed the introduction of the concepts of “computational learning models,” machine learning (ML) and deep learning (DL). AI applications appear promising for radiology scenarios potentially improving lesion detection, segmentation, and interpretation with a recent application also for interventional radiology (IR) practice, including the ability of AI to offer prognostic information to both patients and physicians about interventional oncology procedures. This article integrates evidence-reported literature and experience-based perceptions to assist not only residents and fellows who are training in interventional radiology but also practicing colleagues who are approaching to locoregional mini-invasive treatments.

## 1. Introduction

The term “artificial intelligence” (AI) includes computational algorithms that can perform tasks considered typical of human intelligence, with partial to complete autonomy, to produce new beneficial outputs from specific inputs [1]. Although premises to the development of AI were achieved in the early era of computers, it has only been with the introduction of new powerful computational hardware, in association with the capability of collecting and storing huge amounts of data, that it has become feasible to explore its potential in tasks most relevant to the field of radiology such as pattern recognition, pattern identification, planning, language comprehension, object and sound recognition, problem solving, prognosticating diseases, and deciding when and whether therapy is not needed or of limited use or in offering patients and physicians prognostic data on treatment outcomes. Indeed, although healthcare represents a challenging field for AI application, medical imaging is currently one of the most promising areas to apply this technology [2].

From the beginning, it has been quite clear that computers could be potentially useful in assisting the radiologist in the routine tasks of detection and diagnosis. The idea fostering the use of the so-called computer-aided detection/diagnosis (CAD) systems, precursors of modern AI, was to provide radiologists with the assistance in the detection and interpretations of potential lesions (especially in mammography and chest or musculoskeletal radiography) in order to discriminate between benign and malignant lesions, reduce false negatives, and boost radiologists' productivity, especially in terms of discovery and identification of significant findings requiring a prompt human validation [3]. Main limitations of CAD systems were their task-specific orientation which is suited to only one particular given task in a corresponding specific imaging modality and, moreover, their reliability and the risk of false positive results implied mandatory validation by a trained radiologist [3]. Since then, ever-increasing attempts have been made to improve upon the diagnostic performance of AI and facilitate the help it could provide in daily clinical practice.

The development of AI is largely based on the introduction of artificial neural networks (ANN) in the early 1950s [4] and their subsequent further evolution (from single to multilayer ANN), introducing the concepts of “computational learning models,” machine learning (ML) and deep learning (DL).

ML is based upon the so-called “reverse training” method, in which computer systems focus on specific pathological features identified during a training period [5]. Thus, ML applications require a set of data on a specific pathology on which the computer can train itself, and those data must necessarily contain the desired outcome that needs to be predicted (e.g., nodules or emphysema on chest X-rays, focal liver lesions, hemorrhage in head CT, and so on). Big data is the type of data that may be supplied into the analytical system so that an ML model could learn, improving the accuracy of its predictions. Once trained, the computer can apply this information even to new cases never seen before [6, 7]. ML can be supervised or unsupervised, depending, respectively, on the “labeled” input previously selected by human experts, or directly extracted by the machine using several computational methods [6, 8]. Among the evaluated features, the ideal ML model should include those most relevant to the outcome and the most generic ones which can be applied to the general population, even though it may not be possible to identify these features beforehand. Typical ML tasks in radiology are the *identification* of specific patterns/conditions or *image segmentation*, which can be defined as the representation through partitioning of the digital image into meaningful parts (i.e., pixels or segments) for interpretation. Both have been successfully applied over a wide range of clinical settings including for the detection of fatty liver using ultrasound [9], CT carotid plaque characterization [10], and prediction of lesion-specific ischaemia from quantitative coronary CT angiography [11].

A significant step forward is represented by deep learning (DL), which is based on the implementation of a large number of ANN layers, allowing determination of more complex relationships (similar to neuronal networks) and a more sophisticated performance, attributes particularly suited for imaging. More important, DL is able to perform higher level classification tasks and to automatically extract and learn features, which is valuable when managing the information content of digital images that are only partially detectable and usable by a human reader. This concept unveils the extraordinary potential of DL in comparison with conventional imaging management.

The presence of numerous neural layers between input and output and the use of several techniques (most commonly called convolutional neural networks—CNN) contribute to the plasticity of DL and offer the potential to mimic human brain mechanisms in the training process. Crucial to success of the method is the exposure of CNN to data, in particular images, which can be processed during “training” (supervised or unsupervised). If data are unlabeled, the learning process is based on the automatic clustering of image findings according to their natural variability. Hybrid learning models that include some human guidance are most often used, due to the difficulty of successfully achieving truly unsupervised training. DL represents a hot topic in research, literally exploding in the last years.

Matching ML/DL image processing with clinical and when available pathological/histological data, to correlate intrinsic diagnostic patterns and features of a CT or MRI scan to a specific pathology and histological subtype, has opened a new window in research establishing so-called radiomics [12–14]. In this setting, CAD can also be taken to a higher performance level. ML-based CAD can be taught on the intrinsic differences of a population and then detect and/or diagnose the variations of a single lesion, allowing the identification of common as well as uncommon cases [15].

Supervised and unsupervised learning are largely based on statistical algorithms [16], with important differences between them. Supervised learning deals primarily with classification (i.e., identification of categories for new observations using the same collected on labelled training data sets) and regression (i.e., predictions on continued variables for new observations inferred on training sets). Unsupervised learning cannot take advantage on the labelling process and manages unclassified data; therefore, recognition of latent patterns is performed by applying clustering (aimed to define groups within data) and dimensionality reduction [16]. The sense of such a classification needs a subsequent validation to assess its utility.

Whichever the ML technique used, each approach presents advantages and disadvantages. General pros have to be considered for ML ability to process large volumes of data, to identify trends and patterns only partly detectable by humans, to face with complexity (multidimensionality of data), and to perform high computational tasks.

These advantages are not without cons. First, huge data sets are necessary to train ML machines, whose collection has been limited for a long time in healthcare (although the development of large databases in the era of the so-called “big data” is going to be more widespread). But even when available, the “quality” of data is a major challenge both for the supervised training (due to the large amount of effort needed for labelling data) and the unsupervised training (process of selection and validation).

Moreover, ML assessment represents a critical aspect in terms of statistical power definition (sensitivity, specificity, error susceptibility, and so on) of ML within the task (especially in clinical settings), often in the absence of “disclosure” about “how and why” machines elaborate their tasks, which raises problems when ML applications are introduced in routine medical activity [1, 2, 6, 8, 16].

The aim of this article is to integrate evidence-reported literature and experience-based perceptions, while attempting to make the information easy to access, assisting not only residents and fellows who are training in interventional radiology, but also practicing colleagues who are attempting to gain further expertise with these locoregional mini-invasive treatments.

## 2. AI and Interventional Radiology

### 2.1. Treatment Response

AI applications appear promising for radiology scenarios, as they naturally affect and potentially improve upon lesion detection, segmentation, and interpretation of imaging—prerequisites for good interventional radiology (IR) practice [17]. Moreover, advantages are foreseen even in areas previously not addressed.

One of the biggest challenges of interventional radiology is to estimate/forecast the outcomes and/or the benefits of a treatment before actually performing it [18]. The identification of an accurate method to predict the success rate of a specific treatment in a specific patient could reduce unnecessary and useless procedures and interventions, reducing healthcare costs and dramatically decreasing the risk for the patient. It should also be useful to investigate how a patient's demographic and pathologic characteristics before the treatment can influence treatment efficacy, which can then be measured with posttreatment evaluations.

This type of challenge can be readily taken up using AI and DL, using a computer which autoimproves itself by learning from given inputted data. A patient's baseline diagnostic images, clinical data, and characteristics and outcomes of the planned intervention can be retrospectively applied to a cohort of patients to teach the computer to construct and work on a model that can correlate and “learn” the relationship between those model variables and procedural results. The resultant refined model would then allow the prediction of the procedural outcome in future new patients even before performing the procedure, assuming the characteristics of the intervention are specified. Classification of patients as a responder (complete or partial) or nonresponder could potentially be used in daily clinical practice as an indicator to decide whether or not a specific intervention should be performed [19]. DL-based prediction models can assist interventional radiologists in making decisions as to what procedure will offer the best outcome for each patient. Obviously, these prediction models would require a continuous evaluation and validation to limit or even eliminate possible errors and improve performance in both terms of diagnostic and therapeutic efficiencies.

The field of interventional oncology could greatly benefit from AI, given the great variety of data on which the prediction for daily clinical practice can be made, even though there is the need for more data to help implement ML in the best way [18]. A robust and trustworthy perspective on procedural outcomes could give interventional radiologist more and more solid data upon which to recommend a particular and specific treatment to each patient. In particular, Abajan et al. evaluated the capacity of artificial intelligence to predict chemoembolization outcomes in patients with hepatocellular carcinoma, based on baseline magnetic resonance imaging, dividing patients into responders and nonresponders. They obtained a very good negative predictive value (88.5%) based upon the ML models that relied upon the two features of tumour signal intensity and the presence or absence of cirrhosis [19]. In another anatomic site, the brain, Asadi et al. performed studies on prediction of procedural outcome in stroke and brain arteriovenous malformations patients and successfully individualized treatment based on predicting features [20, 21]. Nonetheless, even if AI can provide information on disease and treatment correlation, it does not necessarily provide an insight on causality and pathophysiology; this information can be, however, obtained from randomized controlled trials, making these two approaches complementary to each other, to design the best treatment strategy.

### 2.2. Procedural Guidance and Support

Owing to the evolution of ML/DL, we are currently surrounded by technology to such an extent that it can assist us, among other tasks, to overcome distances and grant access to extensive knowledge. Touch and touchless devices are everywhere, simplifying our life in many ways, from phone and home assistants to intelligent lights or thermostats, to smart-locks and navigators, and with the introduction of sharing platforms and networks, streaming channels, and live-chat channels as well, our world can be seen as a great, unique web of people.

In an operating room setting, and more specifically in the interventional radiology suite, one of the most important things in procedural planning is the assessment of the patient's anatomy and its pathophysiologic changes. There is also much other valuable information archived in online databases or literature, ranging from (1) individual patient characteristics such as those on tumour characteristics and behaviour which are useful in the specific field of oncological interventions; (2) evidence to support or overcome a particular and unforeseen problem or finding; and (3) local hospital information on angio suite supplies, on the availability of specific devices such as a microcatheter, guidewire, or metallic coils. Currently, however, in large part but not exclusively due to sterility issues, procedural information must be collected beforehand, in the preprocedural planning, whereas, during the procedure, the interaction between the operator and the great amount of patient, literature, and supply data can only be achieved through sterile covers, or indirectly made by other team members, which implies a certain amount of distraction, errors, and time consumption. Nevertheless, these obstacles could be overcome with the implementation, in medical clinical practice, and particularly in operatory theaters and angio suites, of touchless interaction devices, ranging from eye-tracking systems to inertial sensors, to cameras or webcams, to voice-driven smart assistants [22].

Gesture-capture camera systems, with or without utilization of inertial sensors, have been experimented with defining and associating specific actions to a variety of gestures to control medical image viewers while in surgical scrub [23–25]. Indeed, voice recognition interfaces have been demonstrated to enable significant time sparing when dealing with switching on and off operating room components [26]. Navigation systems constructed using inertial sensors worn under sterile gloves have been tried for needle insertion path planning, with a claimed gesture-recognition rate of 100% for 3/4 gestures [27]. Augmented reality devices, such as glasses, which interactively display to the operator the whole variety of relevant information or diagnostic images have also been tested [28, 29].

A group of researchers from the University of California, San Francisco, tested the possibility to question a smart assistant—previously instructed with a large database of information on sheath sizes and compatibility—to obtain suggestions as to which sheath is likely to be most appropriate for the deployment of a particular endovascular stent, during a specific interventional procedure, without removing the sterile surgical scrub, with good results both in terms of time sparing and accuracy [30].

As in the above-mentioned case, questions regarding the correct size of a device or on the time-consuming task of assess for the availability of a particular device or instrument according to the hospital stocks could be directly and instantaneously answered by the smart computer. Questions to the smart assistant could also imply a cost analysis, allowing the operator to choose between two devices not only assessing their dimensions but also their expensiveness in relation to outcome data, providing to all angio-suite staff the perception of the real global cost of a procedure, which must not be taken lightly, minimizing the waste and the inappropriate utilization of guidewires, catheters, coils, and other devices [18].

## 3. Future Perspectives

Most researchers agree that the future of AI lies in enhancing and assisting interventional radiology, not taking over from interventionalists.

*Augmented reality*, in which additional information about the patient can be provided to the operator in real time during the operation, is another technology already being put into practice. When this is combined with machine learning, the algorithm could help the radiologist to make more rapid proper and accurate decisions in terms of diagnosis, treatment management, and planning. Earlier diagnosis through quicker, more accurate reading of scans might enable cancer to be detected earlier, enabling treatment at an earlier stage, with less need for invasive standard surgical approaches. Collaboration between computer algorithms—with their ability to synthesize and spot patterns in vast data sets—and skilled operators—who are able to make sense of the “messiness” of the human body by arriving at correct conclusions despite the multiplicity and complexity of the situation—could raise the standard of IR across the board. Yet, there are significant challenges to overcome before these technologies can be considered mainstream. Regardless, currently, there is intense enthusiasm on the part of clinicians who are calling for increased collaboration between computer scientists, biomedical engineers, and interventional radiologists as machine learning is posited to play a more prominent role in interventional radiology procedures, from informing the initial diagnosis to patient selection and intraprocedural guidance.

## 4. Conclusions

The emerging role of AI may offer the opportunity to better tailor treatment to patients according to “big data” that can be rapidly analyzed, uncovering new insights that may otherwise have required decades of prospective trials. Thus, this new approach could most likely result in a paradigm shift in the near future, definitively changing the current conventional treatment algorithms of tumour therapy, providing superior really personalized care to patients.
